# Leaving safety to visit a feeding site: is it optimal to hesitate while exposed?

**DOI:** 10.1098/rsos.160910

**Published:** 2017-01-11

**Authors:** Sean A. Rands

**Affiliations:** School of Biological Sciences, University of Bristol, Life Sciences Building, Tyndall Avenue, Bristol BS8 1TQ, UK

**Keywords:** optimal foraging, feeders, starvation/predation trade-off, travelling, distance to cover, refuges

## Abstract

Animals living in complex environments experience differing risks of predation depending upon their location within the landscape. An animal could reduce the risk it experiences by remaining in a refuge site, but it may need to emerge from its refuge and enter more dangerous sites for feeding and other activities. Here, I consider the actions of an animal choosing to travel a short distance between a safe refuge and a dangerous foraging site, such as a bird leaving cover to visit a feeder. Although much work has been conducted examining the choice between a refuge and a foraging site when faced with a trade-off between starvation and predation risk, the work presented here is the first to consider the travel behaviour between these locations. Using state-dependent stochastic dynamic programming, I illustrate that there are several forms of optimal behaviour that can emerge. In some situations, the animal should choose to travel without stopping between sites, but in other cases, it is optimal for the animal to travel hesitantly towards the food, and to stop its travel at a point before it reaches the refuge. I discuss how this hesitant ‘dawdling’ behaviour may be optimal, and suggest further work to test these predictions.

## 1. Introduction

The threat of being eaten influences many of the behavioural and life-history decisions made by organisms [1,2]. Animals can adjust the predation risk that they experience by choosing where they move. Seeking and remaining within sheltered locations will reduce exposure to predators [3,4], but animals using these refuges will face a trade-off between being safe and being able to conduct other mutually exclusive behaviours such as foraging or seeking a mate. Because it will need to eat before it starves, an animal will be expected to dynamically switch its behaviour between seeking refuge and foraging [5,6].

Many environments are heterogeneous, and an animal will experience differing feeding opportunities and levels of predation risk as it moves through the landscape [7–10]. In some environments, such as patchy woodlands, an animal may be able to move quickly back and forth between exposed foraging areas and refuge areas where it is safe from being attacked, minimizing its overall exposure to predators [11,12]. Previous studies have explored what animals should do when they have to break cover and visit a foraging site [11–17], where the space that lies between these two distinct regions is ignored. This means that little consideration has so far been given to the behaviour that animals show when moving through the exposed region between the refuge and a feeding site.

Continuously travelling back and forth means that an animal is spending periods of time neither in the safety of its refuge nor at a foraging site where it is able to replenish its diminishing energy reserves. The exposed region between the two sites is itself unlikely to be homogeneous in the risks that the animal will be exposed to [8,9]. Even if there is a constant risk of a predator attacking within the region, an animal close to the refuge at an intermediate location is likely to have a reduced overall chance of being predated when a predator appears, as it is more likely to reach cover than an animal that is further away and that has to travel a longer distance to safety. So, it could be the case that being close to a refuge is nearly as safe as being in the refuge, which in turn means that the animal has to spend less time in travelling a shorter distance between the intermediate semi-safe location and the foraging site. So, should animals stop at intermediate points in this exposed zone when they are travelling between safety and a foraging site?

I explore the decisions an animal makes during this travel period using stochastic dynamic programming [18–21], a technique where it is possible to consider the effects that multiple consecutive behavioural decisions have upon the fitness of an animal in a given state. I use the technique to identify the optimal choice of movement for an animal that has to move away from safety and travel through a risky environment towards a feeding site, and where the animal's metabolism means that it is spending energy continuously. The animal therefore faces a trade-off between staying under cover and potentially starving to death, or risking travel to the foraging site and exposing itself to predation. The trade-off between starvation and predation is well characterized [5,6] and has been explored within dynamic programmes (e.g. [19,22–25]), but although some consideration has been given to animals choosing to explicitly change location between a safe refuge and a dangerous foraging site [5,26] (rather than an implicit assumption that they are doing this by altering their behaviour), no consideration has been given to how the animal should behave in the dangerous exposed region between these two locations, where the danger is as great as being at the foraging site. Here, I describe a simple model of this travel behaviour through the exposed region, and explore whether there are general qualitative predictions about this behaviour that could be used to inform further work. The framework I present is particularly suitable for experiments where feeders are set a short distance from cover (e.g. [15,27–29]), and I give suggestions about how the model could be extended to consider these.

## 2. Material and methods

### 2.1. Model outline

An animal is assumed to exist in an environment consisting of a foraging site located *D* units away from a safe refuge, separated by *D* − 1 exposed intermediate points. At any given moment in time *t*, the animal is found at location *d* (one of these *D* + 1 locations). Any changes to the environment or the animal occur at discrete time-steps. If the animal is in its refuge, it is presumed to be safe from predators, but if it is outside the refuge, there is a probability *α* that a predator appears during a time-step. If a predator has appeared, the animal is killed with probability *π* per time-step. If a predator appears, it is assumed that it will immediately pursue the animal until it has either escaped or been captured. The animal is therefore assumed to start to flee homeward in the next time period, moving one step closer to the refuge at each decision period until it has entered the refuge. The predator remains present in the environment until the animal has returned home, meaning that an animal that encounters a predator close to the refuge suffers fewer periods with the risk of being predated, when compared with an animal closer to the foraging site. Once the animal has returned home, the predator immediately departs. I note here that I am not considering predation risk as being different for a foraging animal and for a travelling animal (although it would be interesting to extend the model to consider how increased exposure while foraging affects decision-making). I also note here that because I am not explicitly modelling predator spatial location, I am therefore ignoring considerations about flight initiation distance [30–32]. I do however consider that the energy reserves of an animal will add to its predation risk through mass-dependent costs [33], where individuals with higher energy reserves will incur a greater risk. These costs are modelled by considering an exponent of the individual's energy reserves (as conducted in Bednekoff & Houston [34]), multiplied by a scalar *σ*. A larger value of *σ* implies a higher risk of being predated with large energy reserves. So, if a predator is present, I consider the probability of an exposed individual being killed during a period to be *π *+ *σx*^2^.

If no predator is present in the environment during a time period (or no predator has yet appeared), the animal can choose between three options (denoted *u*, the distance moved towards the foraging patch): move one step towards the foraging patch; remain in its current location; or move one step back towards the refuge at *d* = 0. For most locations, all three of these behaviours are possible. However, if the individual is at the home location, it can only choose between *u* = 0 or +1, whereas if it is at the foraging patch, it can only choose between *u* = −1 and 0.

The individual can only increase its energy reserves when it is at the foraging site, where it gains energy at a mean rate $\bar{\gamma }$ (with known variance: the individual has a set probability (*γ*_0_, *γ*_1_, *γ*_2_) of finding 0, 1 or 2 energy units of food when it is foraging). Because the model considers an individual that is assumed to be active within the environment, I assume here that it is metabolically active in this model regardless of whether it is moving or not, and it is assumed to have a mean metabolic rate $\bar{\kappa }$ that does not differ with activity conducted (where the metabolic rate also has a known variance, with the individual having a set probability (*κ*_0_, *κ*_1_, *κ*_2_) of losing 0, 1 or 2 energy units in a period).

Given these assumptions, optimal policies were calculated using stochastic dynamic programming [18–21]. Full details of the model are given in appendix A.

### 2.2. Forward simulation

For each optimal policy generated, sets of 1000 individuals were run independently through each policy for 20 000 time-steps. Each individual within a set started at *t* = 0 in the refuge *d* = 0, with initial energy reserves sampled from a uniform distribution of (1, 2, …, *x*_max _− 20) energy units. The period's decision was taken from the optimal policy, and metabolic expenditure (and energy gain if appropriate) were randomly generated according to the parameters that generated the policy. In the forward simulations, predation did not occur, and so the forward simulations solely consider the behaviour of an animal following an optimal policy in a risky environment. Any deaths occurring are therefore due to the animal starving to death. The parameter space chosen was sufficient to ensure that most (99.87 ± 0.008%, mean ± s.d.) of the individuals within each simulation set survived through to the end of their forward runs.

Summary statistics were then calculated as the mean value of the following measures for each set of those individuals who were still alive at the end of a forward run. The two state values of each individual were tracked over time to calculate its mean energy reserves and proportional mean distance from the refuge, and its initial departure time was recorded as the period that it first moved away from the refuge. Once an individual had left the refuge for the first time, the time spent in the refuge after its initial departure was recorded for the remaining periods, as well as the number of visits made to the refuge and the length of each visit. Also recorded were the time spent foraging, the number of visits to the foraging site and the length of foraging episode (noting that an individual forages when it spends a period static at the foraging location), the time spent static when at an exposed location in between the refuge and foraging site, and the number of stays at exposed turning points (taken to be a location where the previous change in location was homeward, and the next change in location was towards the foraging location). If an individual was static during the last (20 000th) period of a simulation, additional periods were calculated solely to assess whether this was a minimum turning point or not—these additional periods did not contribute to any of the other metrics calculated.

Individuals that are not at either the home site or the foraging site during a period are considered to be moving either outwards or homewards. An individual is classified as moving outwards if its last non-zero movement was towards the foraging site, and homewards if its last non-zero movement was towards the home site. These two classifications mean that individuals that have been static for one or more periods are still considered to be in directed transit, based on their last movement. The speed when moving towards and away from the foraging site of an individual are the proportion of those periods where the individual is classified as moving in a given direction where its location changes between consecutive time-steps.

### 2.3. Model exploration

In total, 50 000 policies were calculated for independently generated parameter sets, as detailed in appendix B, keeping the foraging site at 80 units from the refuge. Models were coded in C++, using a Mersenne twister algorithm for random number generation.

To examine the effects of distance, 5000 parameter sets were also generated, and policies were calculated for these while systematically altering the distance of the foraging site to be 20, 40, 60, 80 and 100 units from the refuge.

Using the three policy classifications described in the results section (identified from initial pilot simulations), summary statistics were explored and visualized using *ggplot2* [35] within R v. 3.2.2–4 [36]. To explore whether policy form had an effect on the summary statistics collected, permutation tests were conducted [37,38]. For each summary statistic, *F*_obs_ was calculated as the standard *F*-value from one-way ANOVA (where d.f. = 249 997 for all measures except outward speed, where d.f. = 249 996 through one datapoint being corrupt, and length of home visit where only those simulations where individuals returned home were considered, using d.f. =22 420). Corresponding *F*-values were calculated for 10 000 complete permutations of the entire dataset, and from these sets the largest value was identified, denoted ‘*F*’_max_, corresponding to significance levels of *α* = 0.0001. Sensitivity analyses were also conducted exploring the interaction between the policy classifications and parameter values. The results of these are presented in the electronic supplementary material, figures S1–S11, but are not discussed in any detail here as the focus of this work is on the policy forms generated by the model.

## 3. Results

**Figure 1.**
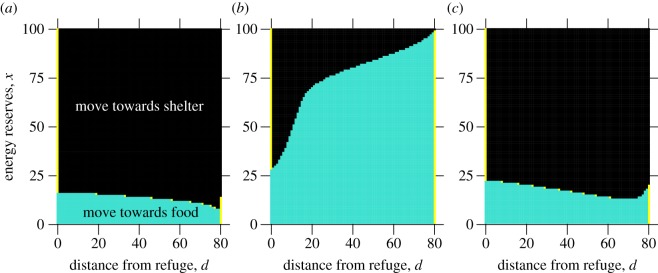
Examples of the forms of policy produced by the foraging model. Each panel shows the single optimal behaviour when a predator is absent from the environment, calculated for all possible energy reserves *x* at all possible distances *d* from the refuge at *d* = 0, where foraging occurs at *d* = 80. If a point is black, the individual should move homewards during the following period; if it is dark turquoise, it should move towards the foraging site; if it is light yellow, it should remain in its current location. (*a*) A *decreasing* policy (*α* = 10^−10^, *γ*_0_ = 0.10, *γ*_1_ = 0.05, *γ*_2_ = 0.85, *κ*_0_ = 0.989, *κ*_1_ = 0.01, *κ*_2_ = 0.001, *π* = 0.001, *σ* = 10^−12^); (*b*) an *increasing* policy (*α* = 0.001, *γ*_0_ = 0.3, *γ*_1_ = 0.1, *γ*_2_ = 0.6, *κ*_0_ = 0.94, *κ*_1_ = 0.05, *κ*_2_ = 0.01, *π* = 0.01, *σ* = 2 × 10^−10^); (*c*) an *intermediate* policy (as *b* but *α* = 10^−5^, *π* = 0.001, *σ* = 10^−12^).

Optimal policies were generated when exploring the parameter space described. In all the policies (figure 1), the animal should move away from home towards the foraging site if its energy reserves are very low (as it would otherwise be at risk of starvation), and it should always move back towards home if its reserves are suitably high (as it is unlikely to starve, and so is exposing itself to unnecessary predation risk). The policy forms differed in how individuals with intermediate levels of energy reserves should behave, mostly showing a clear energy threshold level at a given location, such that the animal should move homeward if its reserves fall above this threshold, and move to the foraging site if its reserves fall below the threshold (although it is optimal to not move at some of these threshold values, as shown by the yellow points on figure 1). This essentially means that the individual should tend to move towards the foraging site when its energy reserves are suitably low, and towards the refuge once it has replenished its reserves, and so following the policy should keep the individual within the state space in the policies that is close to the switching threshold between behaviours.

Three forms of policy emerged from parameter exploration. In the first (figure 1*a*), labelled a *decreasing* policy, the threshold describing the switch between behaviours falls monotonically as distance from the refuge increases. In the second (figure 1*b*), labelled an *increasing* policy, the threshold increases monotonically as distance from the refuge increases. Note that the example shown in figure 1*b* is an extreme example—not all increasing policies rise such that the threshold at foraging site is the maximum energy reserves. The final policy form, labelled an *intermediate* policy (figure 1*c*), has a non-monotonic threshold that falls and then rises again as distance from the refuge increases, giving an intermediate location where the threshold is lowest.

In all the policy types shown, the animal should remain in the refuge if its energy reserves are above the threshold (shown by the yellow bar on the left side of each diagram). When the animal is at the foraging site, it should stay if its reserves are below the threshold, but it should be noted that for the decreasing policies, this switching threshold at the foraging site can be higher than the switching threshold at the location immediately before the foraging site (seen as the jump between the turquoise ‘move towards food’ region and the right-hand yellow region in figure 1*a*). This means that once the animal has reached the foraging site, it should build its energy reserves by staying for a number of periods before it moves off.

Having identified these three policy forms, the data generated were sorted according to the optimal policies the simulated individuals were following. This was done for each policy by identifying the lowest energy value at each location at which the optimal behaviour switched, and then identifying whether these threshold values increased, decreased or showed an intermediate minimum with respect to distance from the refuge. For the policies generated, 11.9% were decreasing, 29.3% were increasing and 58.8% were of the intermediate form (table 1). For the fitness values calculated by the dynamic programme for optimal policies (electronic supplementary material, figure S12), calculated fitness tended to increase sigmoidally with distance from the refuge when predators were absent, and decay with a decelerating form when predators were present. All else being equal, individuals with low energy reserves had lower calculated fitnesses.

**Table 1.** The effects of the policy types on the measured statistics (shown with standard deviations, where appropriate). Policies were classified according to the descriptions given in the Results. Also given are indications of how statistics change in response to increasing the distance between the refuge and foraging location, summarizing the trends given in the electronic supplementary material, figures S13–S15. For these distance summaries, ‘↓’ indicates a reduction, ‘↑’ indicates an increase, ‘∪’ indicates a minimum value falling at a distance falling between the shortest and longest distances considered, ‘∩’ indicates a similar maximum and ‘—’ indicates no directional relationship.

	increasing		decreasing		intermediate	
	policy	distance	policy	distance	policy	distance
number of simulations generating policy	14 655		5958		29 387	
initial departure time	1526.3 ± 1479.4	↓	2017.8 ± 1568.2	∪	1385.5 ± 1324.1	↓
number of visits back to refuge	26.7 ± 20.8	↓	1.7 ± 4.3	↓	6.5 ± 15.1	↓
time spent at refuge after initial departure	13 613.5 ± 3282.1	↓	207.5 ± 637.8	↓	821.7 ± 2107.7	↓
number of simulations with individuals returning to refuge	14 655 (100.0%)		1167 (19.6%)		8411 (28.6%)	
length of visit to refuge	1293.3 ± 1474.6	↑	112.3 ± 65.4	∪	111.8 ± 86.5	↑
number of visits to foraging site	27.6 ± 22.0	↓	274.3 ± 872.4	↓	95.8 ± 119.9	↓
time spent foraging	521.5 ± 291.2	—	329.2 ± 222.0	↓	524.6 ± 286.7	—
length of foraging episode	31.9 ± 22.4	↑	3.5 ± 2.1	∩	6.1 ± 2.7	↑
proportional mean distance from refuge	0.14 ± 0.09	↑	0.73 ± 0.13	↑	0.64 ± 0.16	↑
speed when moving towards foraging site	1.00 ± 0.00	—	0.26 ± 0.23	↑	0.32 ± 0.17	↑
speed when moving away from foraging site	1.00 ± 0.00		1.00 ± 0.00^a^		1.00 ± 0.00	
time spent static and exposed	2.7 ± 26.7	—	12 777.4 ± 2799.0	↓	10 545.8 ± 3468.2	↓
number of exposed turning points	0.7 ± 2.5	∪	272.5 ± 872.7	↓	89.1 ± 122.7	↓
energy reserves	43.75 ± 16.99	↑	16.64 ± 2.31	↑	18.71 ± 3.52	↑

^a^All variation in speed to the refuge seen happened for this policy, where 52 individuals stopped at least once on their way back to the refuge; for the other two policies, all individuals travelled at top speed.

**Figure 2.**
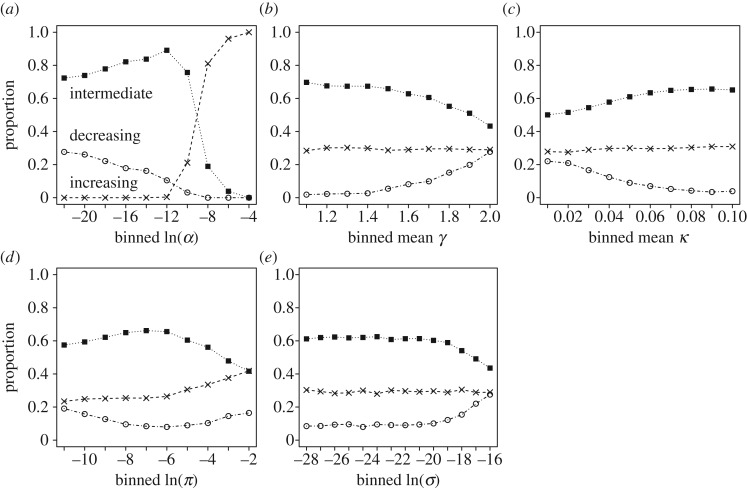
Effects of the model parameters on the form of policy produced. Parameters randomly generated for model exploration were continuously distributed, and are presented here by presenting the proportion of each policy type calculated for binned sets of parameter values (where the reported parameter represents the upper limit of the bin), shown for: (*a*) probability of predator appearing, *α*; (*b*) mean energy gain ($\bar{\gamma }=\gamma_{1}+2\gamma_{2}$) when foraging; (*c*) mean metabolic cost ($\bar{\kappa }=\kappa_{1}+2\kappa_{2}$) during a period; (*d*) probability of being killed when a predator is present, *π*; (*e*) mass-dependent predation scalar, *σ*. Empty circles, decreasing policies; crosses, increasing policies; filled squares, intermediate policies.

The form of policy seen was influenced strongly by the environmental predation risk, *α*. In environments with a high chance of a predator appearing (high *α*), policies tended to be increasing (crosses, figure 2*a*). As environmental risk decreased, intermediate policies became more common (filled squares, figure 2*a*), showing a peak in the proportion of policies produced at intermediate environmental risk levels. Although decreasing policies were never the most common policy in the parameter set explored, they did become gradually more common as risk was reduced (open circles, figure 2*a*).

The other parameters explored had less pronounced effects on the proportion of each policy type seen. Increasing the mean gain $\bar{\gamma }$ led to more decreasing policies and fewer intermediate policies (figure 2*b*), while increasing the mean cost $\bar{\kappa }$ had the opposite effect (figure 2*c*). The risk of being killed when a predator was present *π* had a subtler effect, with an intermediate maximum in the proportion of intermediate policies and an intermediate minimum in the proportion of decreasing policies at an intermediate value of *π* (figure 2*d*). Increasing the mass-dependent predation scalar *σ* led to an increase in the proportion of decreasing policies and a reduction in the proportion of intermediate policies (figure 2*e*). The proportion of increasing policies seen were little affected by changes in gain, cost or mass-dependent predation scalar (figure 2*b*,*c*,*e*), but showed a gradual increase with an increase in the risk of being killed when a predator was present (figure 2*d*).

For all the statistics calculated, the *F*_obs_ values obtained from resampling were much greater than the ‘*F*’_max_ values from the sampled distribution (electronic supplementary material, table S1), suggesting that policy type can have large effects on the behaviours being measured.

**Figure 3.**
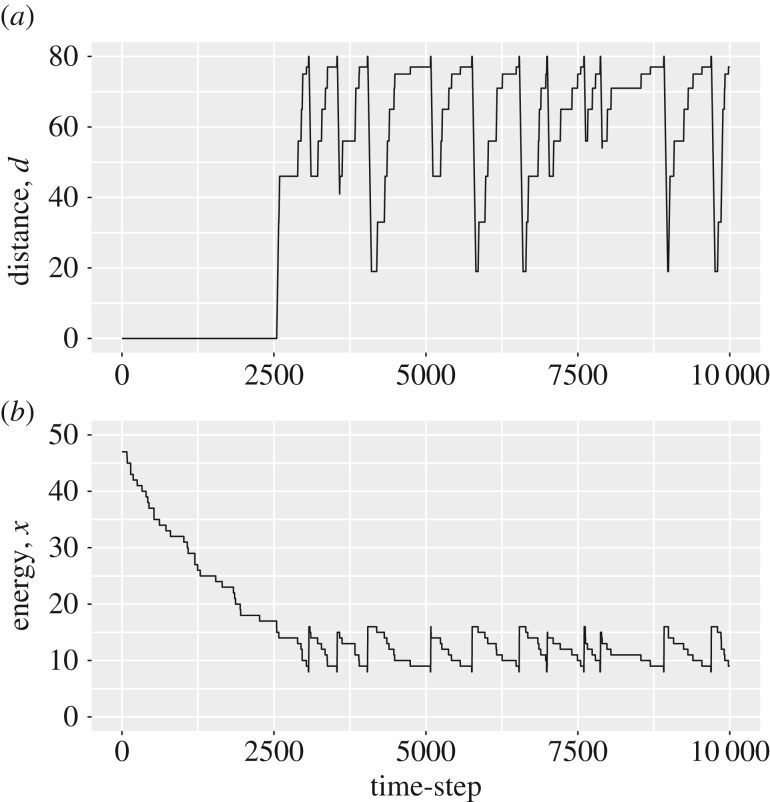
Demonstration of a ‘dawdling’ time series, coming from an individual following the decreasing policy given in figure 1*a*. The distance from the refuge (*a*) and energy reserves (*b*) of an individual are shown for the first 10 000 time-steps of a simulation, where the individual starts with its reserves set at 47 energy units.

Animals following decreasing policies tended to spend a longer time at home before their initial departure, when compared to animals following increasing or intermediate policies (table 1), because the threshold for leaving tended to be lower. Once they had left the refuge for the first time, animals following decreasing policies tended not to return to the refuge often (less than 20% of the simulations following these policies returned home, table 1), but if they did return, they remained in the refuge over multiple consecutive periods. Most of their time was spent nearer to the foraging site than the refuge (table 1). Their movement towards and away from the foraging site followed the form of pattern illustrated in figure 3. Movement towards the foraging site tended to be punctuated by a series of stops and starts, giving a slow mean outward speed (table 1) resulting from a large amount of their time spent static in the exposed region between refuge and foraging site (table 1). Once they arrived at the foraging site, they foraged for a short period of time, and then headed back to the refuge at ‘top speed’, without pausing until they reached an intermediate exposed turning point, whereon they turned and started heading back towards the foraging site (possibly pausing first). This slow movement towards the foraging site and fast movement away was continuously repeated, with the location of the exposed turning point changing over time (as seen in figure 3) as optimal turning behaviour is dependent upon the energetic state of the animal. Individuals following decreasing policies also tended to have low mean energy reserves over time (table 1).

In comparison, animals following increasing policies showed much more time at the refuge, with all individuals returning at some point during the simulations, and spending a much longer time in the refuge per visit than returning individuals following decreasing policies (table 1). They spend very little time static in the exposed region, and tend to shuttle back and forth between the foraging site and refuge, travelling at top speed in both directions and tending not to turn back in the exposed region. As they spend more time at the refuge than foraging, over time they tend to be close to the refuge (as seen in the proportional mean distance to the refuge, table 1). The shape of an increasing policy means that an animal leaving the refuge will tend to spend a long time at the foraging site to build its reserves to a point where it then returns home, and this is seen in the larger amount of time spent foraging (table 1). Animals following increasing policies tend to have much higher energy reserves than with other policies (table 1).

Animals following an intermediate policy tended to show behavioural statistics intermediate between the decreasing and increasing policy behaviours, with their overall behaviour more closely matching the form of individuals following decreasing policies (table 1), with intermediate turning points and stuttering movement out towards the foraging site.

Changing the distance to the foraging site affected behaviour. Table 1 shows the patterns of change seen in the summary statistics when this distance was increased (summarizing the electronic supplementary material, figures S13–S15). Policy form had no effect on several of the patterns seen, which were similar for all three policies: the number of visits back to the refuge or to the foraging site and the time spent in the refuge decreased with increasing distance, while the mean energy reserves increased. A further foraging site (modelled by increasing *D*) meant animals tended to spend more time in the refuge during a visit (unless following a decreasing policy with a close foraging site), and tended to spend longer at the foraging site during a visit (again, unless following a decreasing policy with a close foraging site). Animals tended to stop less when travelling towards the foraging site (except where the policy was increasing, when they would have been travelling without pausing regardless of distance).

## 4. Discussion

The model described here demonstrates that when we are observing the behaviour of an animal choosing to move between a refuge and a close foraging site, it may be optimal for an animal to be making decisions about its behaviour while travelling between these two locations. Dependent upon the form of policy followed, the animal may also show slow ‘dawdling’ behaviour on its way towards the foraging site (but should always return fast), as is seen when policies show decreasing or intermediate forms. For the sake of describing the different forms of behaviour seen, I arbitrarily divided policies into three forms, but it may be more correct that they are seen as a continuum, where intermediate policies are similar to decreasing ones, as is evident from the similarity of the report statistics in table 1. The form of increasing policies suggested that distinguishing these from the other forms is sensible, however. These increasing forms are particularly likely when the probability of a predator appearing is high, which means that animals foraging in dangerous environments should tend to travel quickly between the refuge and foraging site, rather than spend extra time exposed.

The model suggests that in some cases (particularly for decreasing policies) it might be advantageous to remain part-way between the foraging site and the refuge, rather than returning completely to cover. Showing exposed turning points means that an individual does not have to travel the full distance between the foraging site and the refuge each time (saving time and energy), while keeping its minimum distance to the refuge suitably small to reduce its overall probability of being captured by a predator if one appears. Within the model, when the animal initially heads homewards after leaving the foraging site, it does not stop until it reaches a turning point. Because the optimal policy is fixed for an individual, the location of the turning point will depend upon both immediate metabolic expenditure and the energy gained in the previous foraging episode, both of which are stochastic. Empirically, this translates to uncertainty in both food supply and metabolic processes, and should be interpreted as an individual being expected to stop at some intermediate exposed location, rather than as an exact prediction about location (and the stuttering stop–start behaviour when moving back to the foraging location should be interpreted similarly). Furthermore, it is likely that stopping points will be heavily influenced by local environmental features (e.g. convenient points to perch, stand or otherwise rest). An animal in the wild may therefore be likely to follow a simpler heuristic than its optimal policy [39], such as ‘after eating, travel homeward and stop at a convenient exposed location, and wait until your reserves fall to a predefined level’.

Despite the potential ease of observing travel to a close foraging site, and the number of studies exploring this form of foraging behaviour (e.g. [11–17,27–29,40–43]), there is little empirical information about this behaviour, and that which exists (e.g. [41], which shows that several species of bird travelled directly between refuge and foraging site without stopping) is anecdotal. Model predictions are accessible to study with wild individuals foraging at feeders, and could even be explored using common garden feeders (e.g. [27,28]) provided a suitable technique for avoiding pseudoreplication is used. The predictions made here about behaviour in response to differing distances between the refuge and foraging site are particularly open to experimental manipulation, and broadly match existing empirical evidence (e.g. [16,29,42]).

Experiments on fish in tanks containing refuges and foraging sites separated by an open space are particularly suitable for testing model predictions (although tank design may directly influence the behaviour observed [44]). Directness of motion between a refuge and a foraging site or latencies in leaving and travelling across an open space were recorded as a report statistic for fish [45,46], while other reports [47,48] describe time spent in a ‘hesitancy zone’ as an area immediately outside a refuge that individuals may pause in (which may be relevant to the current model, although it is possible that optimal pausing could happen in regions beyond any arbitrarily allocated ‘hesitancy’ threshold).

The model presented here considers a single forager in a simple environment where the forager is unable to influence how it detects and responds to the predator. The forager could show vigilance [49,50], which may enhance its ability to detect the predator early (potentially to the detriment of foraging or travel speed). The forager could also decide the distance at which it is best to start fleeing from the predator, and the speed at which it should escape [30–32], or it could reduce its time at the foraging site by carrying food back to the refuge for processing [11,12,41]. The model also assumes the predator leaves immediately once the forager has returned to its refuge, but if the predator remains, the forager may have to wait a length of time until it is (potentially) safe to emerge [51], which may also be dependent upon its current size and metabolic rate [52,53]. Adding temporal changes to the environment, such as through fluctuating food availability or predator presence [5,6], may also affect the foraging behaviour (potentially pushing foraging behaviour to bimodal peaks similar to those predicted for small birds in winter [18,19], although there is experimental evidence [54] suggesting foraging may occur throughout the day). Finally, the forager's social environment may influence its behaviour and risks incurred [24,55,56]. If there are multiple individuals foraging together with dominance hierarchies affecting how they interact, an individual's position within the hierarchy may determine how far it has to go to forage [14,57], or the amount of access it has to a refuge [58]. The modelling framework I outline here could be extended to consider these additional complications, in addition to being parametrized to more accurately represent the biology of a species of interest.

Here, I have described an optimal behaviour akin to latent approach behaviour as an optimal foraging strategy under the risk of predation. Similar patterns are seen in the cognitive sciences in response to a fitness-reducing factor. A slow approach behaviour could be construed as ‘wariness’ or ‘fearfulness’ by an observer [59,60], and in tests exploring cognitive bias, negative underlying emotional states may lead animals to show latency in approaching stimuli [61]. Tests exploring speed–accuracy trade-offs also explore delays in approaching targets [62]. In this study, I demonstrated that this ‘dawdling’ may be an optimal response, and there could be adaptive value to what observers may see as undirected behaviour.

## Supplementary Material

This material contains two files. The first collects the following figures:• Table S1: results of randomisation test• Figures S1-S11: sensitivity analyses• Figure S12: examples of fitness values generated• Figures S13-S15: effects of changing foraging site distance on summary statisticsNote that the sensitivity analyses are presented here as graphs without further in depth discussion of the trends seen, given that the focus of this paper is on the policy forms.

## Supplementary Material

The code used is included as a separate file ‘supplementary_code.zip’, which contains two files suitable for building with a C++ compiler:• main.cpp, which is the main model code• mersenne_twister.h, which contains the Mersenne twister functionThe latter is an amended version of a library distributed under a GNU Library General Public License - please see the file header for details.

## Data accessibility

C++ code for the model simulations described is included in the electronic supplementary material.

## Appendix A. Model details

An individual's state is characterized by: its energy reserves $x\in (0,1,\ldots ,x_{\max})$; its distance from the refuge d∈(0,1,…,D); and whether there is a predator immediately present in the environment p∈(A,P), where *A* denotes an environment where a predator is absent and *P* denotes an environment where there is a predator present. If the individual is at the foraging site, it gains *γ_i_* = *i* units of energy for *i* = 0, 1 or 2 with probability *Γ*(*γ_i_*), where $\sum_{i}\Gamma (\gamma_{i})=1$. During a time period, the individual also spends *κ_j_* = *j* units of energy for *j* = 0, 1 or 2 with probability *K*(*κ_j_*), where $\sum_{j}\text{K( }\kappa_{j}\text{) }=1$. In the exploration of parameter space, mean gain and mean cost are set such that there is an expected net gain when at the foraging site, and a slow decline in reserves when the animal is elsewhere in the environment.

The environment the individual occupies is assumed to be one-dimensional, such that the individual's location in the environment *d* represents the number of steps it would take the individual to return to its refuge at *d* = 0 if it were to travel directly homewards. The refuge at *d* = 0 is assumed to be safe and predator free, while the patch at *d* = *D* is the foraging site, and is the furthest point the individual can travel from the refuge. If an individual returns to its refuge, any predators previously present in the environment are assumed to have departed as soon as the individual has returned home (when *d* = 0).

The individual is assumed to make a series of consecutive decisions about its behaviour at periods [0, *T*], where *T* is sufficiently large to allow for convergence (see below) to occur in less than *T* periods. At a time *t*, the fitness of an individual at a given state combination is denoted *V*(*x*, *d*, *p*, *t*).

The reward function *R*(*x*, *d*, *p*) at *t* = *T* is set as

A 1 $$R(x,d,p)=V(x,d,p,T)=\left\{ \begin{array}{ll} 0 & \text{if}\;x=0,\\ 1 & \text{otherwise}. \end{array}\right.$$

Having set these final fitness values, the following criteria can be used to calculate all values of *V*(*x*, *d*, *p*, *T* − 1), and these can then be used to calculate all values of *V*(*x*, *d*, *p*, *T* − 2), and so forth, using backward iteration.

If a predator is present

A 2 $$V(x,d,P,t)=\left\{ \begin{array}{ll} \sum_{\kappa =0}^{2}K(\kappa )V(\Lambda (x-\kappa ),0,A,t+1) & \text{if}\;d=0\\ (1-(\pi +\sigma x^{2}))\sum_{\kappa =0}^{2}K(\kappa )V(\Lambda (x-\kappa ),0,A,t+1) & \text{if}\;d=1\\ (1-(\pi +\sigma x^{2}))\sum_{\kappa =0}^{2}K(\kappa )V(\Lambda (x-\kappa ),\;d-1,P,t+1) & \text{if}\;d>1, \end{array}\right.$$

where $\Lambda (x)=\text{min(}x_{\max},\text{max(}0,x\text{))}$.

If a predator is absent and *x* > 0, $V(x,d,A,t)=\max_{u}H(x,0,A,t|u),$ where *u* denotes the behavioural options available

A 3*a* $$\begin{array}{rl} H(x,0,A,t|u) & =\left\{ \begin{array}{ll} \text{not considered} & \text{if}\;u=-1\\ \sum_{\kappa =0}^{2}K(\kappa )V(\Lambda (x-\kappa ),0,A,t+1) & \text{if}\;u=0\\ (1-\alpha )\sum_{\kappa =0}^{2}K(\kappa )V(\Lambda (x-\kappa ),\;1,A,t+1)+\alpha (1-(\pi +\sigma x^{2})) & \\ \;\times \sum_{\kappa =0}^{2}K(\kappa )V(\Lambda (x-\kappa ),1,P,t+1) & \text{if}\;u=+1, \end{array}\right. \end{array}$$

A 3*b* $$\begin{array}{rl} H(x,1,A,t|u) & =\left\{ \begin{array}{ll} (1-\alpha )\sum_{\kappa =0}^{2}K(\kappa )V(\Lambda (x-\kappa ),\;0,A,t+1)+\alpha (1-(\pi +\sigma x^{2})) & \\ \;\times \sum_{\kappa =0}^{2}K(\kappa )V(\Lambda (x-\kappa ),0,A,t+1) & \text{if}\;u=-1\\ (1-\alpha )\sum_{\kappa =0}^{2}K(\kappa )V(\Lambda (x-\kappa ),\;1,A,t+1)+\alpha (1-(\pi +\sigma x^{2})) & \\ \;\times \sum_{\kappa =0}^{2}K(\kappa )V(\Lambda (x-\kappa ),1,P,t+1) & \text{if}\;u=0\\ (1-\alpha )\sum_{\kappa =0}^{2}K(\kappa )V(\Lambda (x-\kappa ),\;2,A,t+1)+\alpha (1-(\pi +\sigma x^{2})) & \\ \;\times \sum_{\kappa =0}^{2}K(\kappa )V(\Lambda (x-\kappa ),2,P,t+1) & \text{if}\;u=+1, \end{array}\right. \end{array}$$

A 3*c* $$\begin{array}{rl} H(x,1<d<D,A,t|u) & =\left\{ \begin{array}{ll} (1-\alpha )\sum_{\kappa =0}^{2}K(\kappa )V(\Lambda (x-\kappa ),\;d-1,A,t+1)+\alpha (1-(\pi +\sigma x^{2})) & \\ \;\times \sum_{\kappa =0}^{2}K(\kappa )V(\Lambda (x-\kappa ),d-1,P,t+1) & \text{if}\;u=-1\\ (1-\alpha )\sum_{\kappa =0}^{2}K(\kappa )V(\Lambda (x-\kappa ),\;d,A,t+1)+\alpha (1-(\pi +\sigma x^{2})) & \\ \;\times \sum_{\kappa =0}^{2}K(\kappa )V(\Lambda (x-\kappa ),d,P,t+1) & \text{if}\;u=0\\ (1-\alpha )\sum_{\kappa =0}^{2}K(\kappa )V(\Lambda (x-\kappa ),\;d+1,A,t+1)+\alpha (1-(\pi +\sigma x^{2})) & \\ \;\times \sum_{\kappa =0}^{2}K(\kappa )V(\Lambda (x-\kappa ),d+1,P,t+1) & \text{if}\;u=+1, \end{array}\right. \end{array}$$

A 3*d* $$\begin{array}{rl} H(x,D,A,t|u) & =\left\{ \begin{array}{ll} (1-\alpha )\sum_{\kappa =0}^{2}K(\kappa )V(\Lambda (x-\kappa ),\;D-1,A,t+1)+\alpha (1-(\pi +\sigma x^{2})) & \\ \;\times \sum_{\kappa =0}^{2}K(\kappa )V(\Lambda (x-\kappa ),D-1,P,t+1) & \;\text{if}\;u=-1\\ (1-\alpha )\sum_{\kappa =0}^{2}\sum_{\gamma =0}^{2}K(\kappa )\Gamma (\gamma )V(\Lambda (x+\gamma -\kappa ),D,A,t+1)+\alpha (1-(\pi +\sigma x^{2})) & \\ \;\times \sum_{\kappa =0}^{2}\sum_{\gamma =0}^{2}K(\kappa )\Gamma (\gamma )V(\Lambda (x+\gamma -\kappa ),D,P,t+1) & \text{if}\;\;u=0\\ \text{not considered} & \text{if}\;u=+1. \end{array}\right. \end{array}$$

Backward iteration was conducted until *t*_conv_, where $\max_{x,d,p}|V^{\prime }(x,d,p,t_{\mathrm{conv}})-V^{\prime }(x,d,p,t_{\mathrm{conv}}+1)|\leq 0.00001$, where *V*′(*x*, *d*, *p*, *t*) is *V*(*x*, *d*, *p*, *t*) standardized so that the highest value is set equal to 1 (such that $V^{\prime }(x,\;d,\;p,\;t)=V(x,\;d,\;p,\;t)/\{\max_{x,d,p}V(x,\;d,\;p,\;t)\}$). Since environmental conditions are not explicitly dependent upon time, I assume that strong backward convergence holds [19,63], and so the results described are independent of assumptions made about the reward function. Therefore, I assume that the policy generated in response to *V*(*x*, *d*, *p*, *t*_conv_) is the optimal policy. I note here that none of the optimal policies generated had ambiguous results—all possible state combinations had a single optimal behaviour associated with it.

## Appendix B. Sensitivity analysis parameters

For parameter space exploration, each parameter set was generated using the following criteria, where *rand_n_* is a random variable independently sampled from a uniform distribution (0,1): *α *= 10*E*(−2–8*rand*_1_); *π *= 10*E*(−1–4*rand*_2_);$\;\sigma =(10E(-3-6{\text{rand}}_{3}))/(x_{\max}^{2})$; *Γ*(2) = 0.5 + 0.49*rand*_4_; *Γ*(0) = (1 − *Γ*(2))*rand*_5_; *K*(0) = 0.95 + 0.45*rand*_6_; and *K*(1) = (1 − *K*(0)*rand*_7_.
