# Supplementary material for: Leaving safety to visit a feeding site: is it optimal to hesitate while exposed?
Source: R Soc Open Sci. 2017 Jan 11;4(1):160910. doi: 10.1098/rsos.160910 (PMC5319356; doi:10.1098/rsos.160910)
Supplement: The code used is included as a separate file ‘supplementary_code.zip’, which contains two files suitable for building with a C++ compiler:• main.cpp, which is the main model code• mersenne_twister.h, which contains the Mersenne twister functionThe latter is an amended version of a library distribute [file rsos160910supp2.pdf]

Supplementary Material

This material contains the following information:

- Table S1: results of randomisation test
- Figures S1-S11: sensitivity analyses
- Figure S12: examples of fitness values generated
- Figures S13-S15: effects of changing foraging site distance on summary statistics

Note that the sensitivity analyses are presented here as graphs without further in depth discussion of the trends seen, given that the focus of this paper is on the policy forms.

The code used is included as a separate file 'supplementary_code.zip', which contains two files suitable for building with a C++ compiler:
- main.cpp, which is the main model code
- mersenne_twister.h, which contains the Mersenne twister function

The latter is an amended version of a library distributed under a GNU Library General Public License - please see the file header for details.

	F_{obs}	$'F'_{max}$
Initial departure time	506.9	8.42
Number of visits back to refuge	8866.1	9.61
Time spent at refuge after initial departure	148244.3	9.33
Length of visit to refuge	3067.4	9.52
Number of visits to foraging site	1299.8	8.32
Time spent foraging	1257.1	9.42
Length of foraging episode	23419.5	11.07
Proportional mean distance from refuge	72385.2	9.67
Speed when moving towards foraging site	104516.1	9.26
Time spent static and exposed	78705.2	9.27
Number of exposed turning points	1576.8	8.67
Energy reserves	36195.1	11.01

Table S1. Results of the randomisation tests. All F_{obs} > $'F'_{max}$, suggesting that the results shown are significant – see the Methods for details.

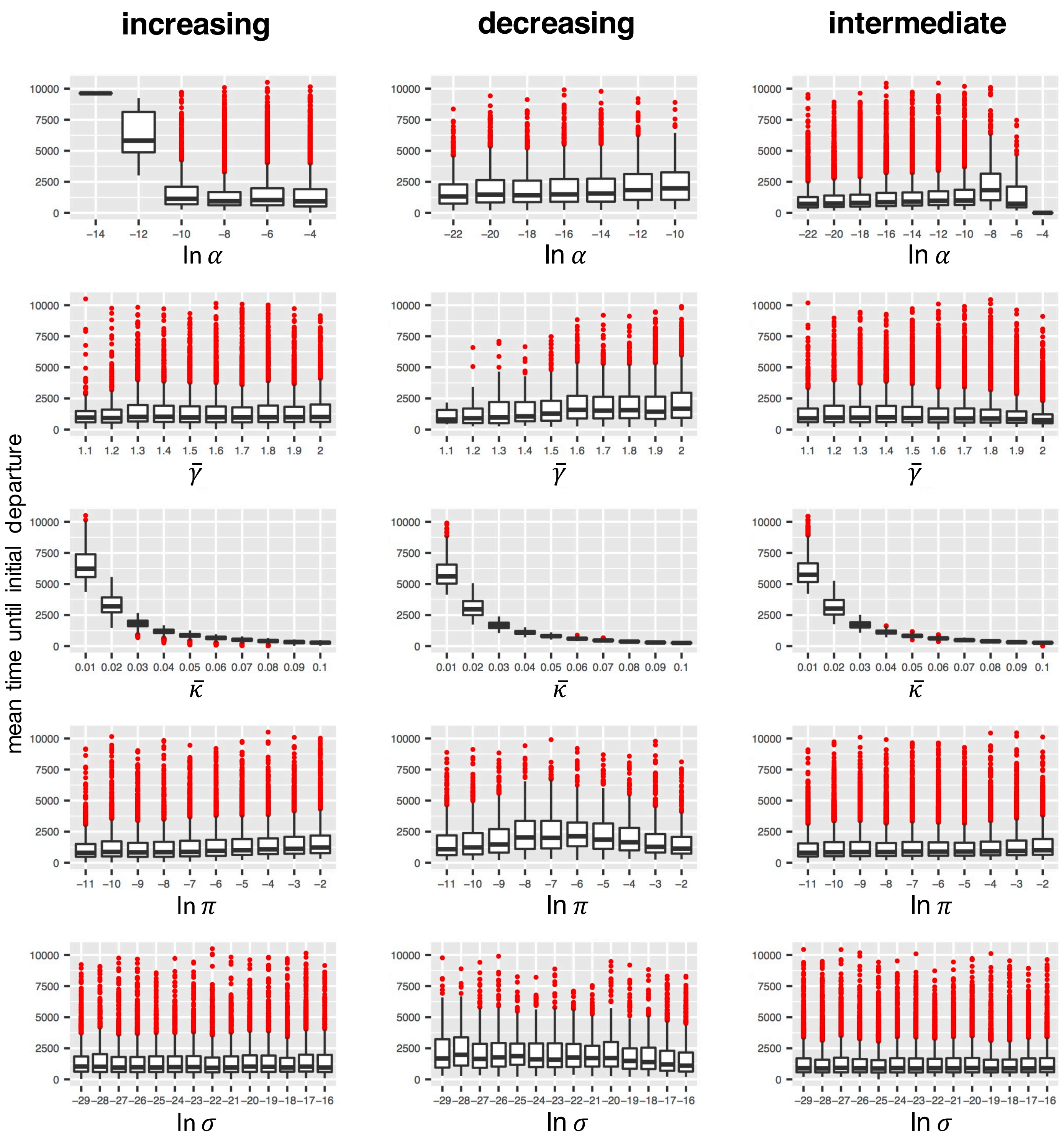

Figure S1. Boxplots of the simulated mean times until initial departure for increasing, decreasing and intermediate policies, summarised according to variation in the five model parameters as described in the methods. Note that the variation in parameters used for generating policies was continuous, but values have been binned together in these figures to make relationships more visible. Boxes represent the median and interquartile range (IQR), and whiskers represent the most extreme value within 1.5 × IQR. Outliers beyond the whiskers are represented with red spots.

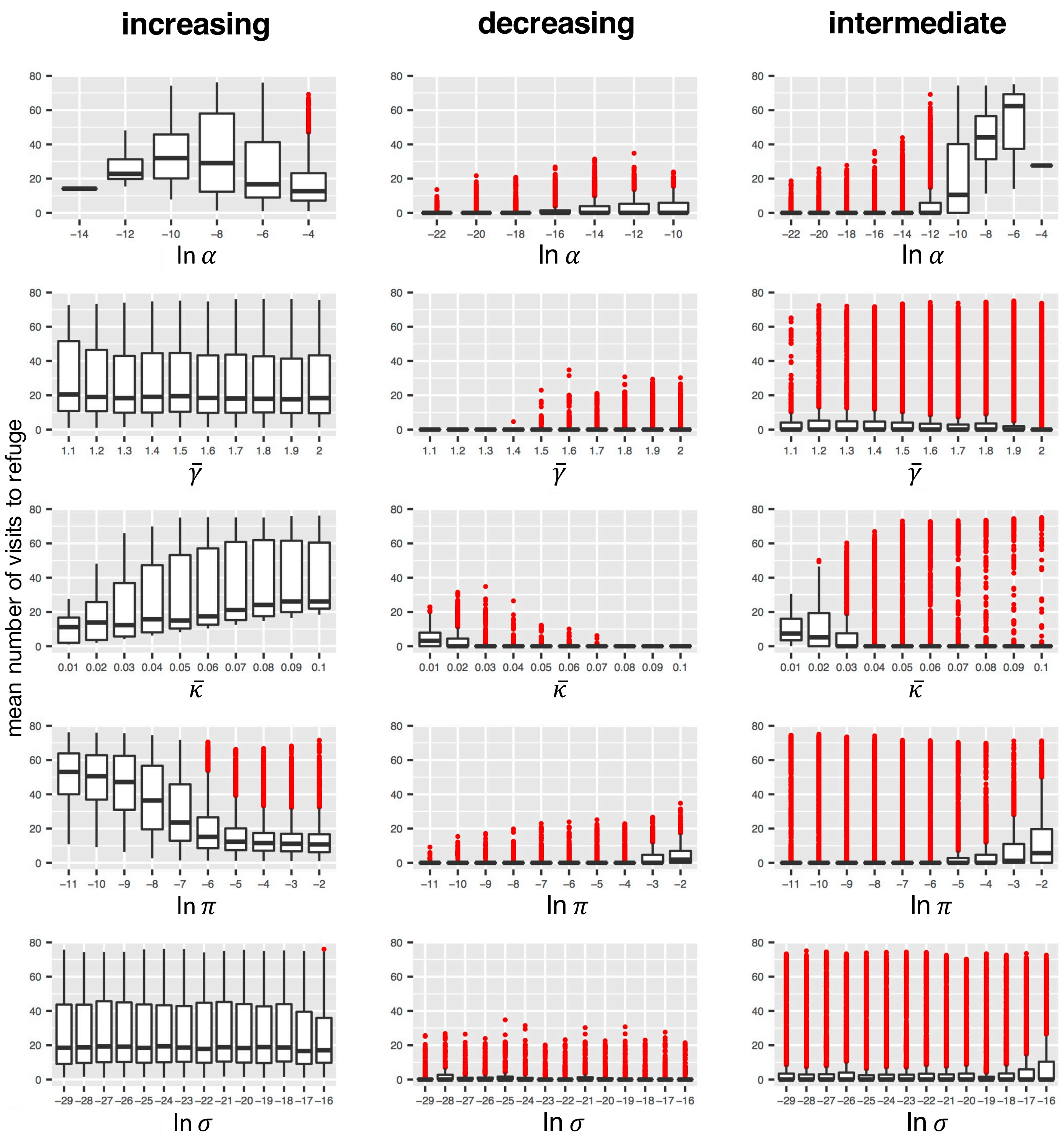

Figure S2. Boxplots of the simulated mean number of visits to the refuge for increasing, decreasing and intermediate policies, summarised according to variation in the five model parameters as described in the methods. See figure S1 for further details.

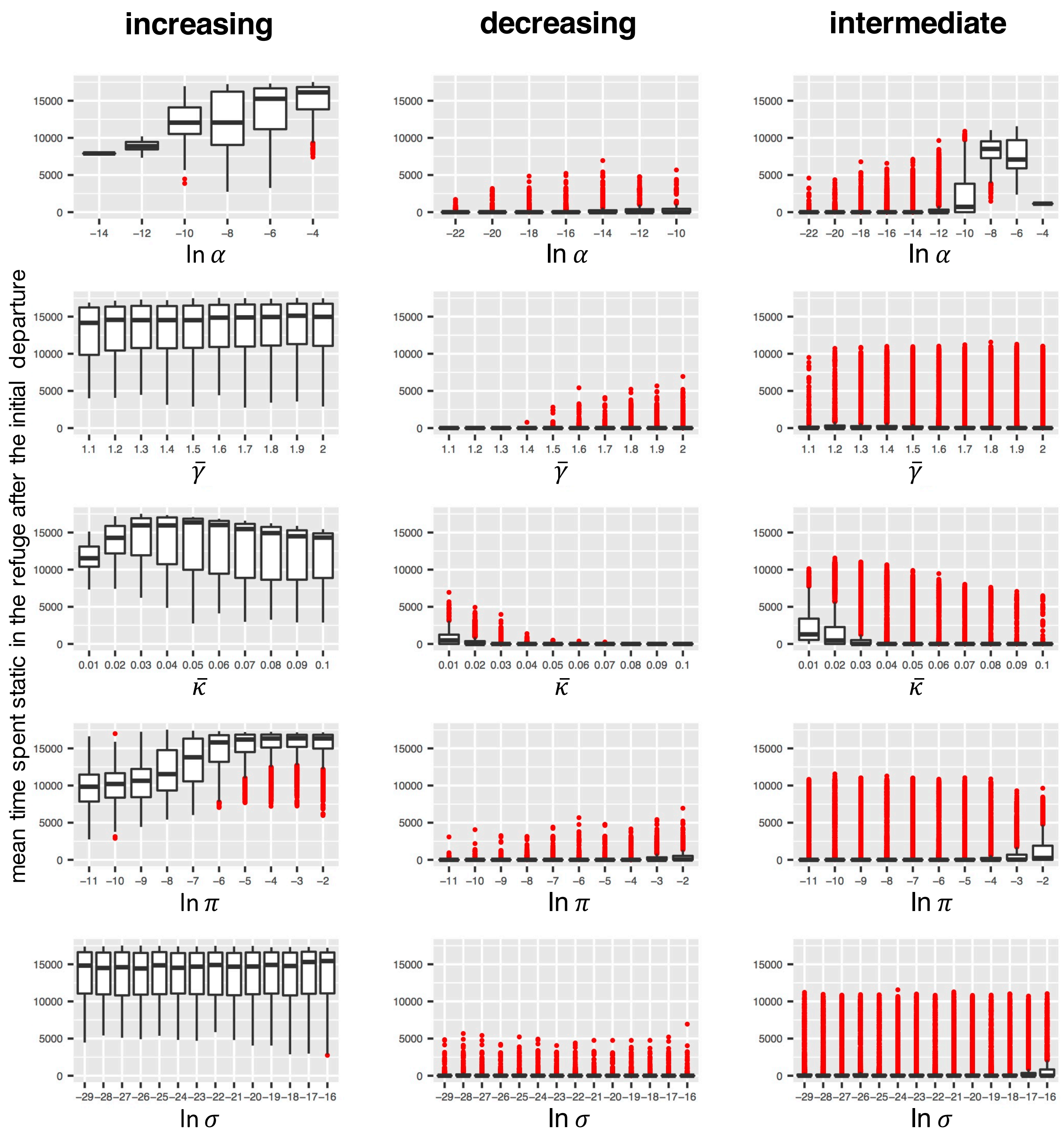

Figure S3. Boxplots of the simulated mean time spent static in the refuge after initial departure for increasing, decreasing and intermediate policies, summarised according to variation in the five model parameters as described in the methods. See figure S1 for further details.

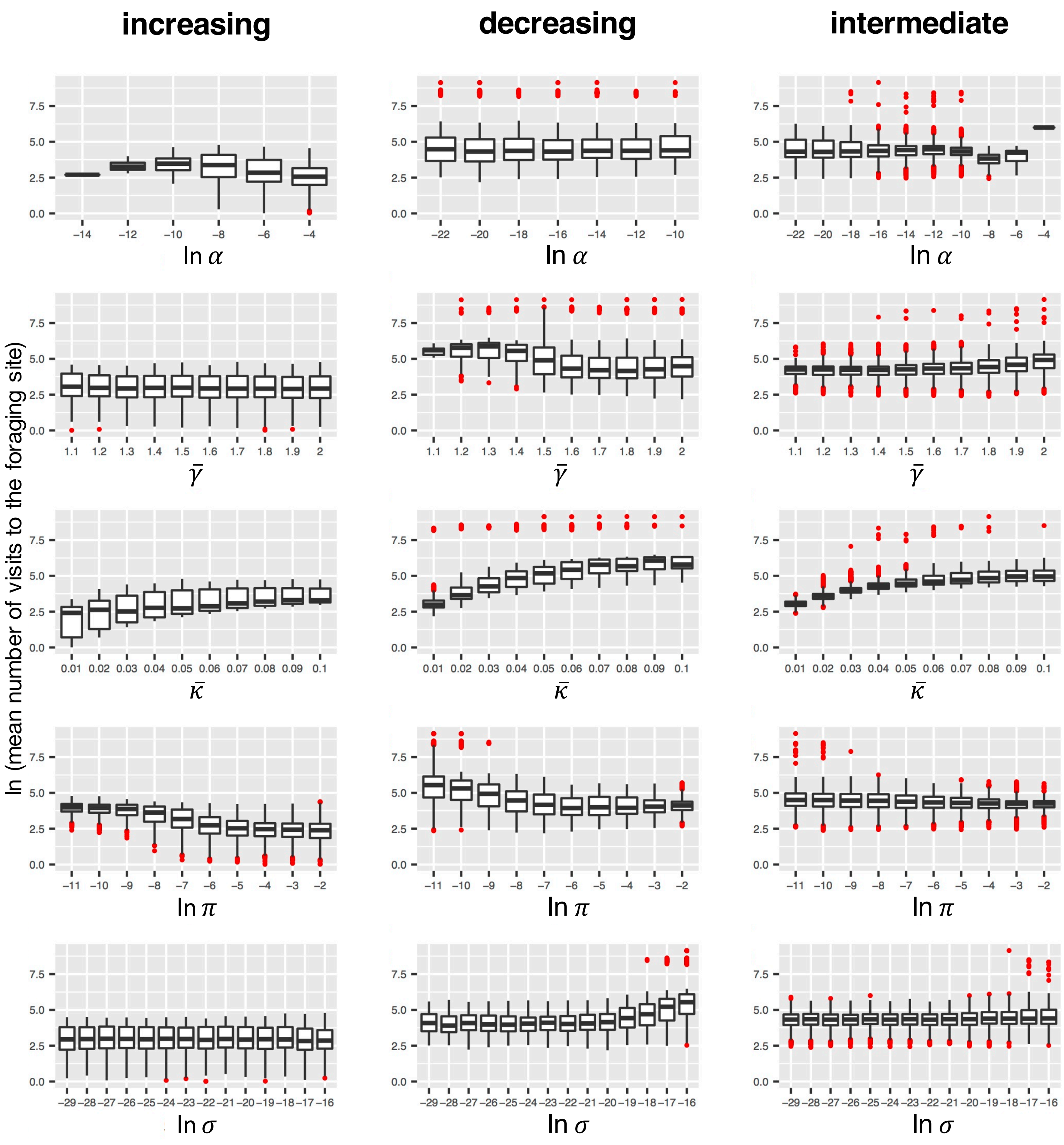

Figure S4. Boxplots of the simulated mean number of visits to the foraging site for increasing, decreasing and intermediate policies, summarised according to variation in the five model parameters as described in the methods. See figure S1 for further details.

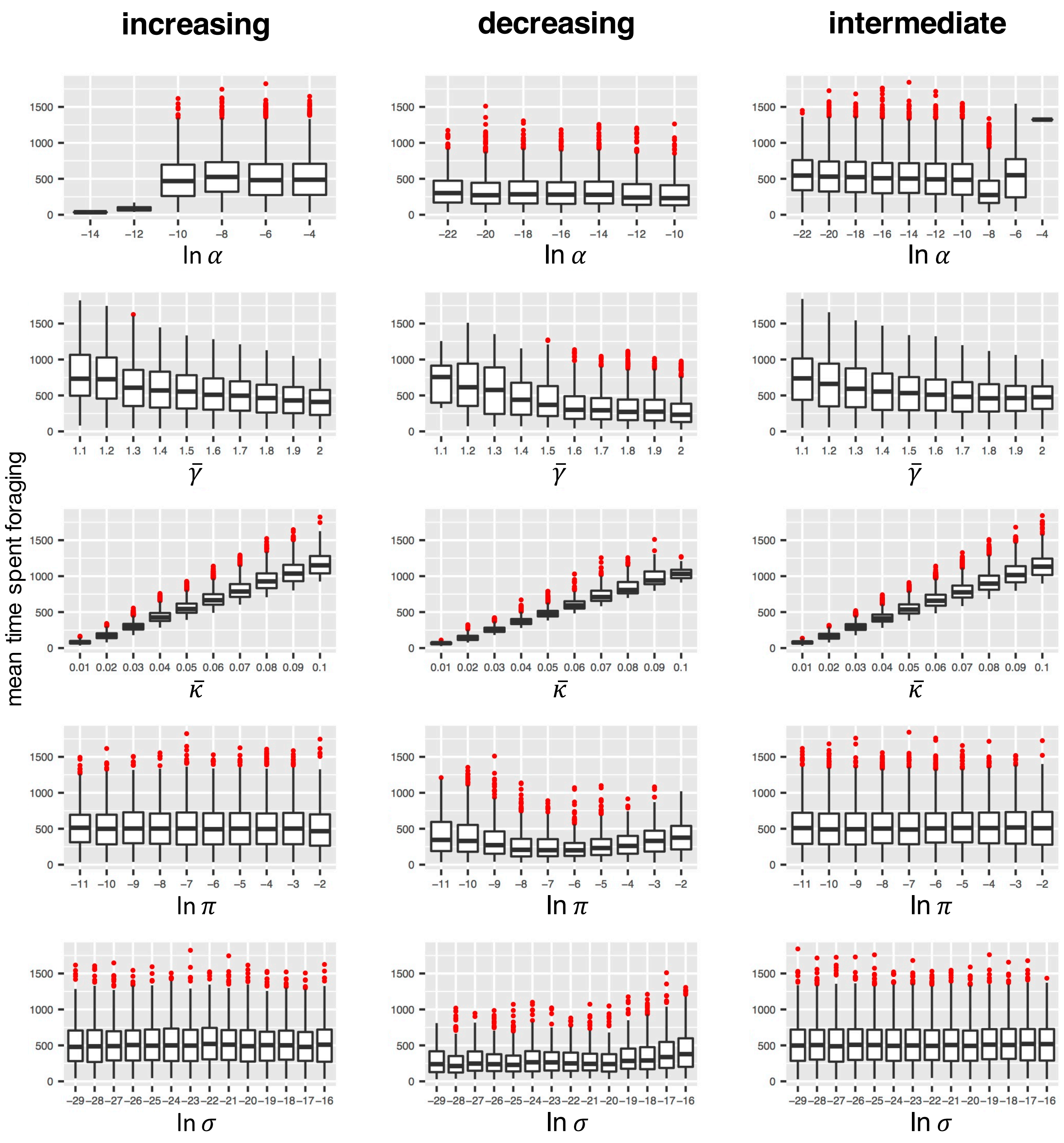

Figure S5. Boxplots of the simulated mean time spent foraging for increasing, decreasing and intermediate policies, summarised according to variation in the five model parameters as described in the methods. See figure S1 for further details.

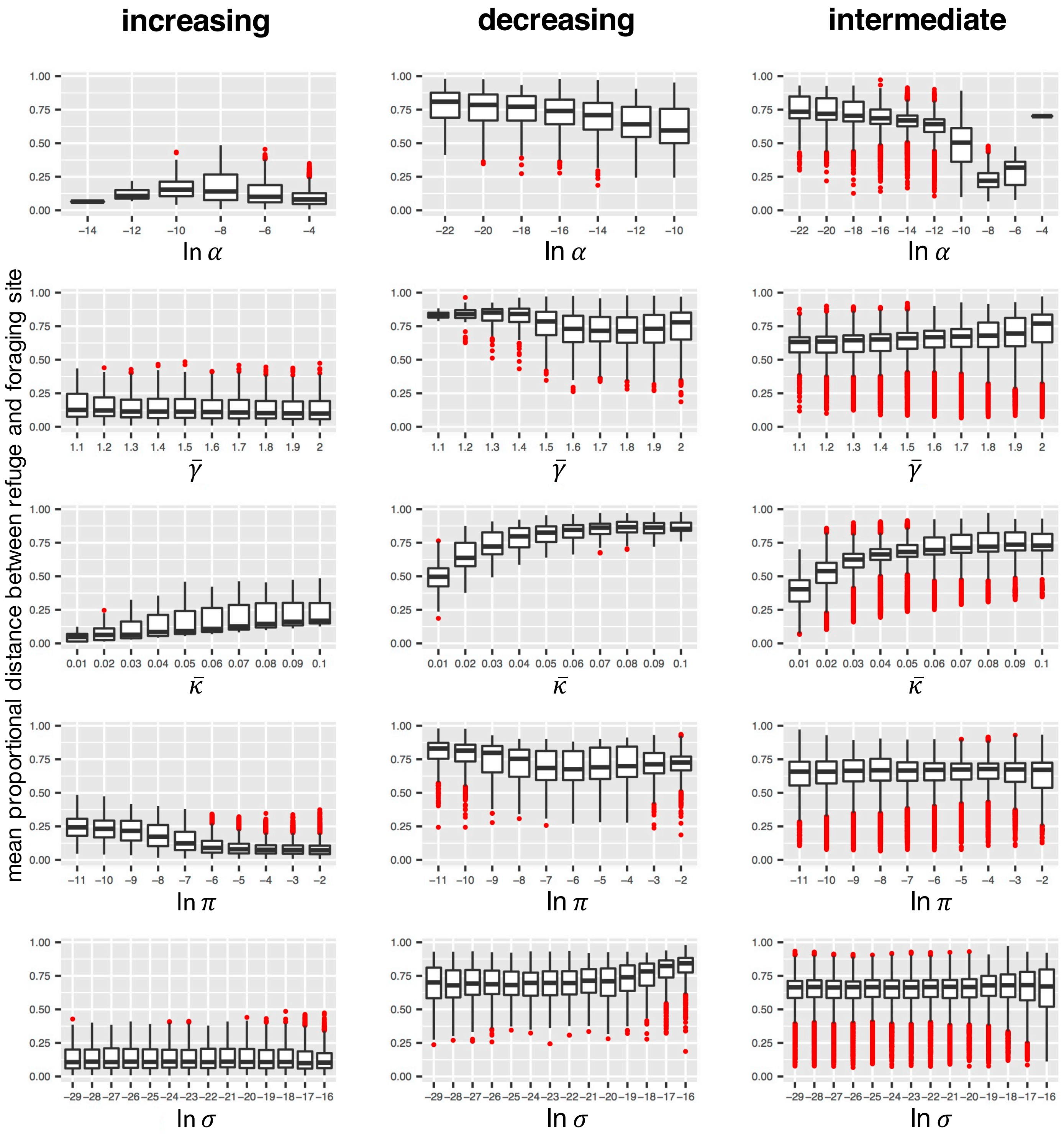

Figure S6. Boxplots of the simulated mean proportional distance of an individual between the refuge and foraging site (where the refuge would be represented by 0.0 and the foraging site by 1.0) for increasing, decreasing and intermediate policies, summarised according to variation in the five model parameters as described in the methods. See figure S1 for further details.

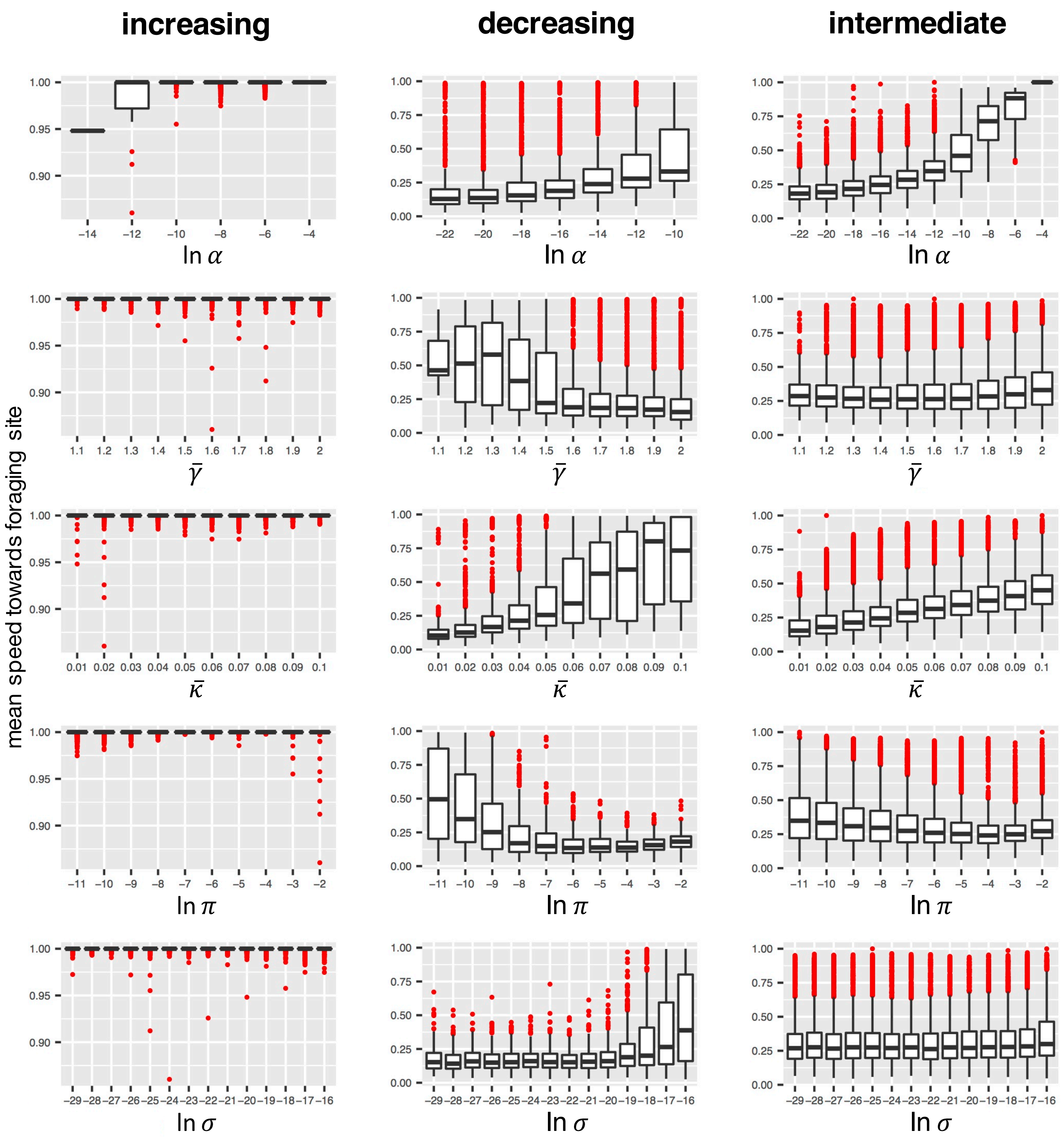

Figure S7. Boxplots of the simulated mean speed when moving towards the foraging site for increasing, decreasing and intermediate policies, summarised according to variation in the five model parameters as described in the methods. See figure S1 for further details.

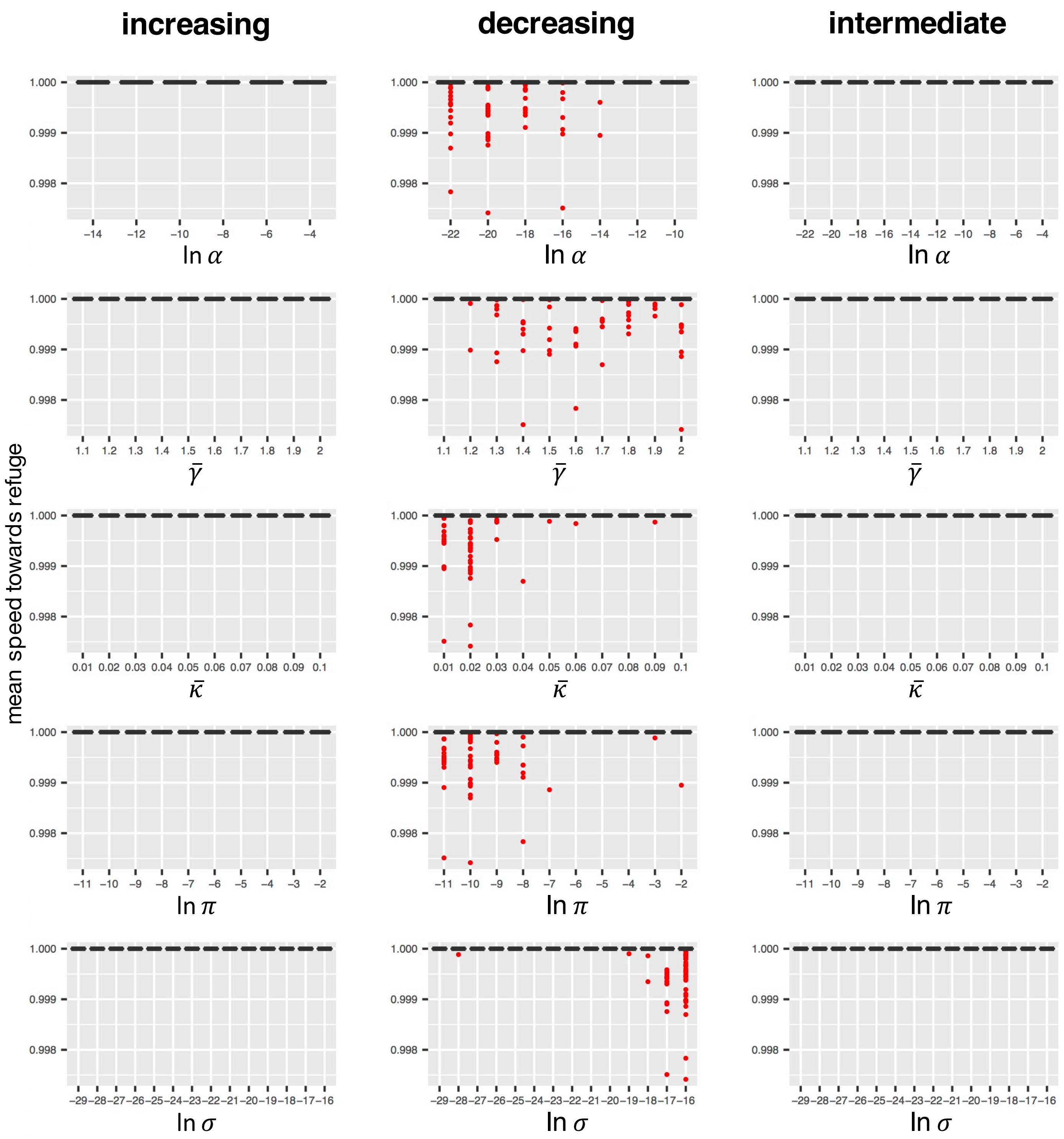

Figure S8. Boxplots of the simulated mean speed when moving towards the refuge for increasing, decreasing and intermediate policies, summarised according to variation in the five model parameters as described in the methods. See figure S1 for further details.

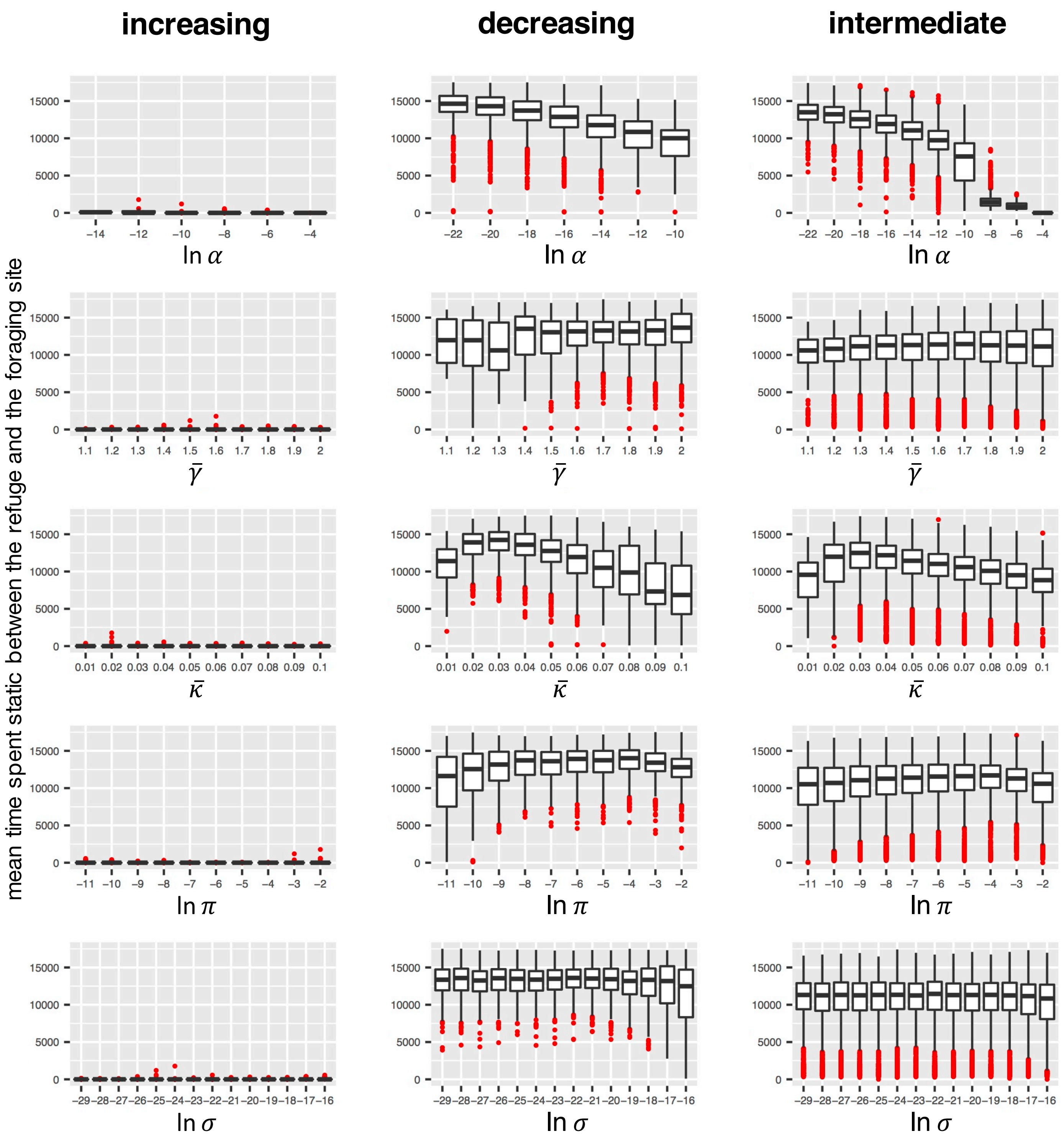

Figure S9. Boxplots of the simulated mean time spent static in the exposed region between the refuge and the foraging site for increasing, decreasing and intermediate policies, summarised according to variation in the five model parameters as described in the methods. See figure S1 for further details.

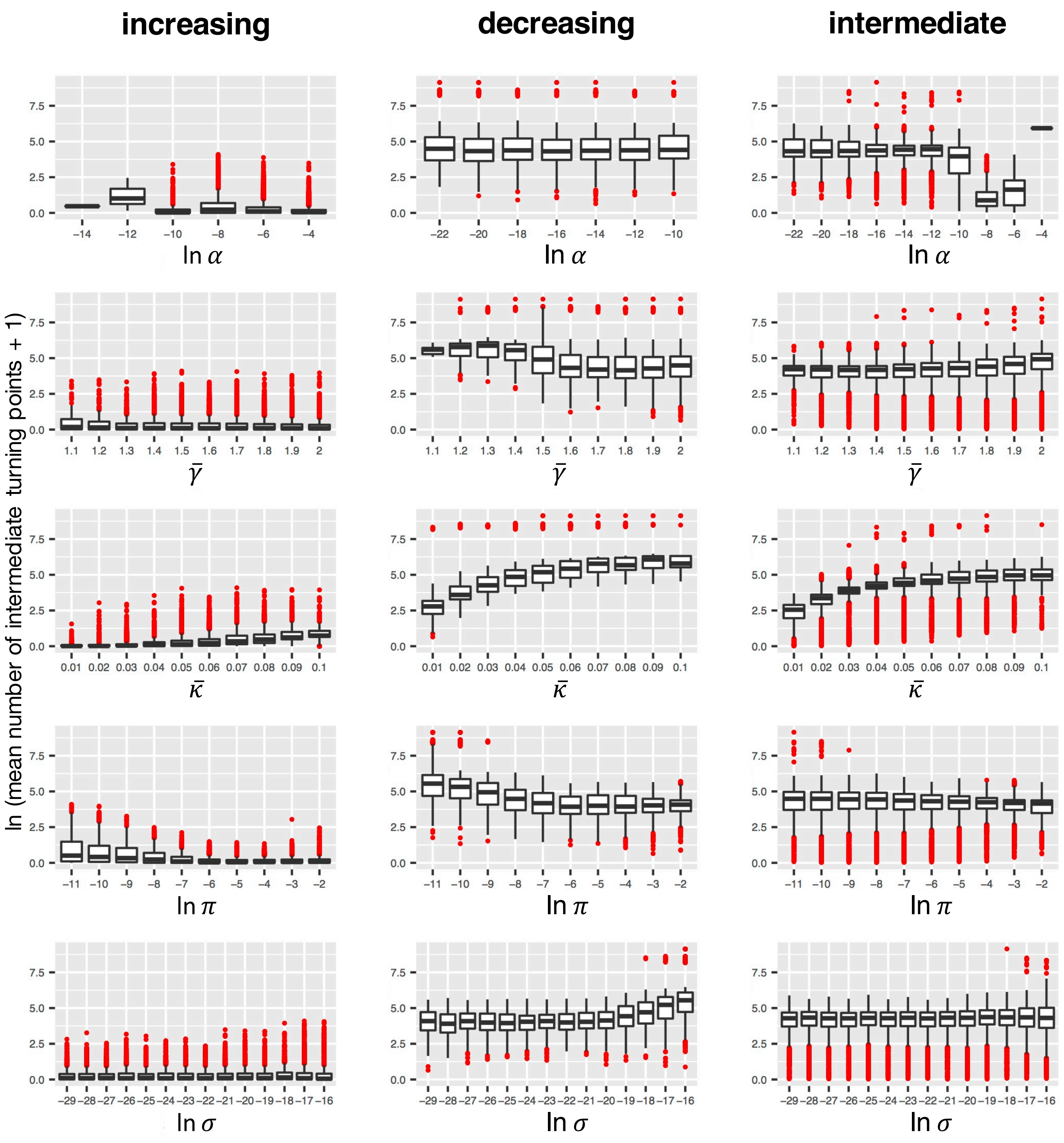

Figure S10. Boxplots of the simulated mean number of turning points in the exposed region between the refuge and the foraging site, for increasing, decreasing and intermediate policies, summarised according to variation in the five model parameters as described in the methods. See figure S1 for further details.

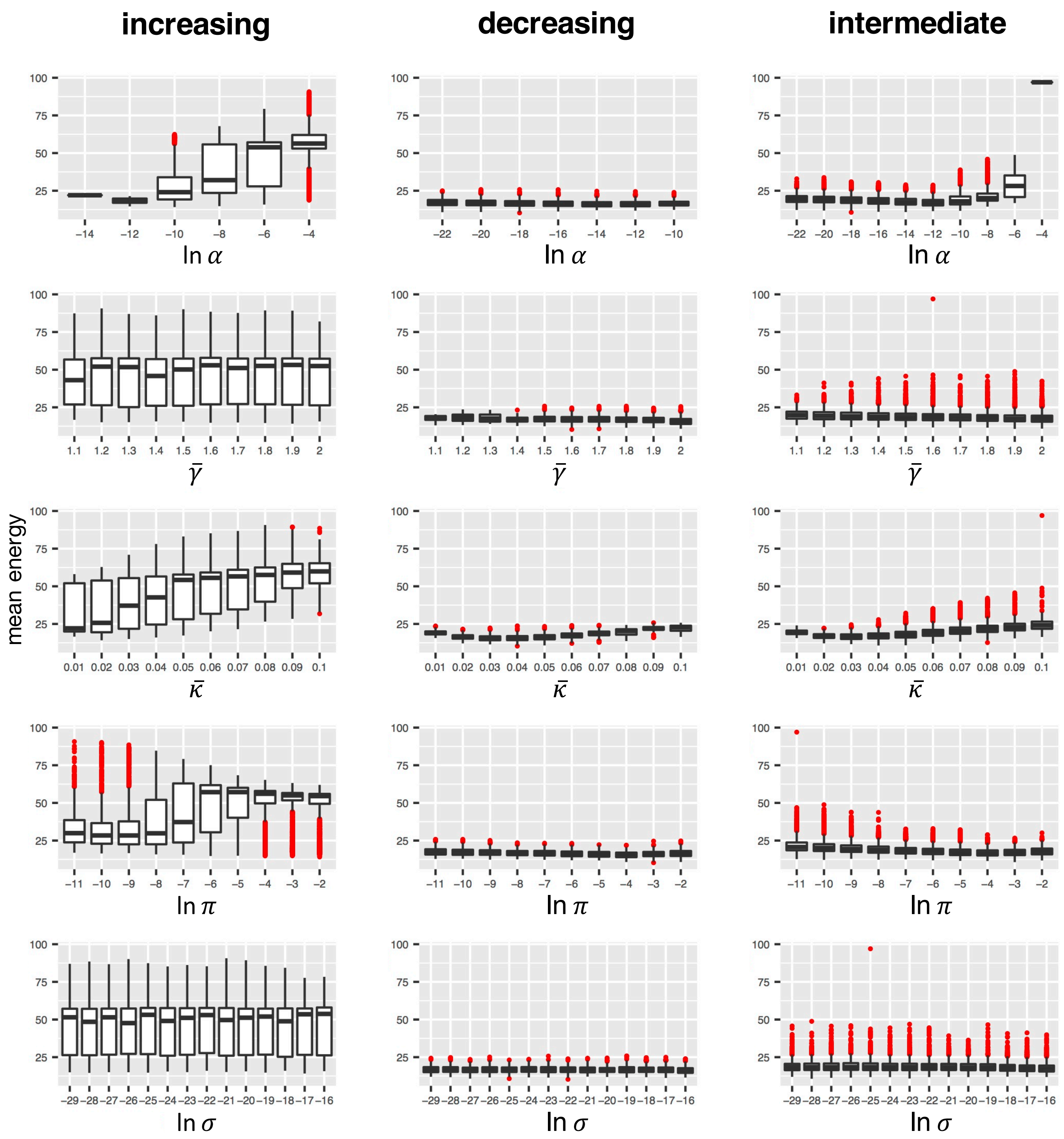

Figure S11. Boxplots of the simulated mean energetic reserves for increasing, decreasing and intermediate policies, summarised according to variation in the five model parameters as described in the methods. See figure S1 for further details.

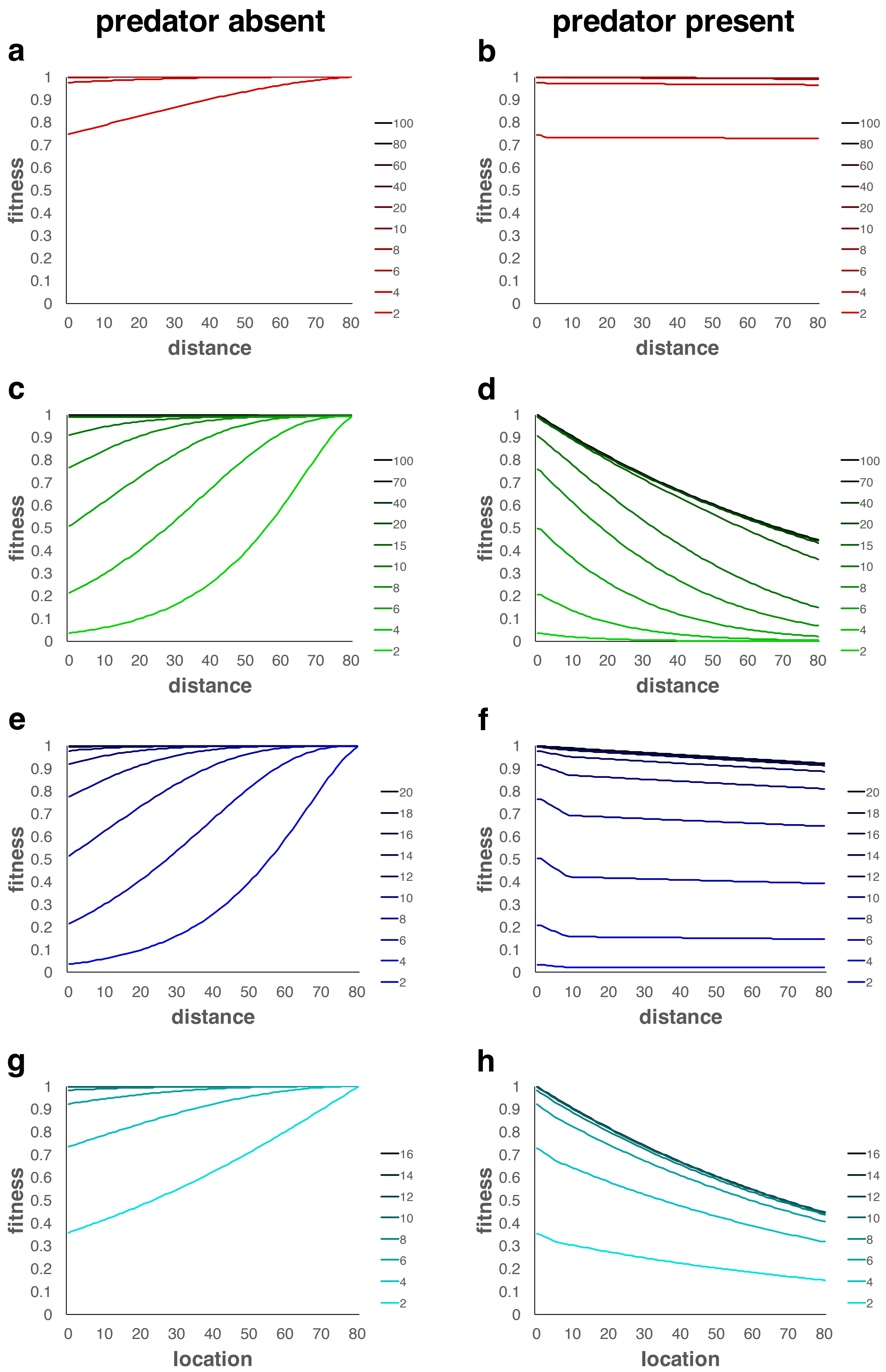

Figure S12. Calculated fitness values for sample optimal policies. Each graph reports fitness relative to location for individuals possessing energy reserves denoted by the labelled colours, where the range of values given for each graph represent the range of differing values seen – larger energy reserves are not reported where there is no visible difference above the largest value given. Left hand figures show fitness when a predator is absent, and right hand figures show fitness when a predator is present, for the decreasing (**a**, **b**), increasing (**c**, **d**), intermediate (**e**, **f**) and complex (**g**, **h**) policies detailed in figure 1.

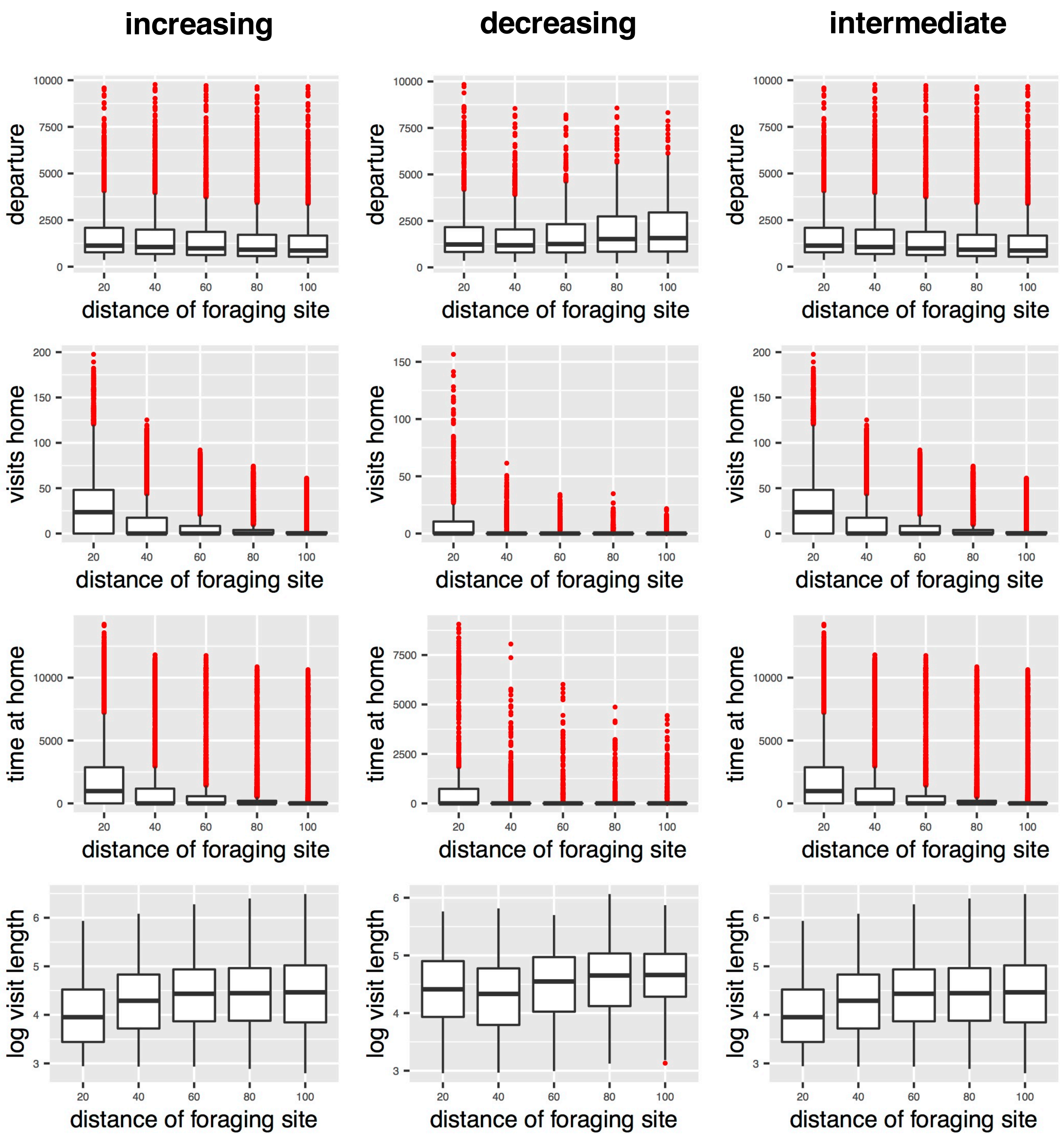

Figure S13. Effects of distance of foraging site on various summary statistics, according to policy form. See panels for details of summary statistic, and see figure S1 for description of boxplots.

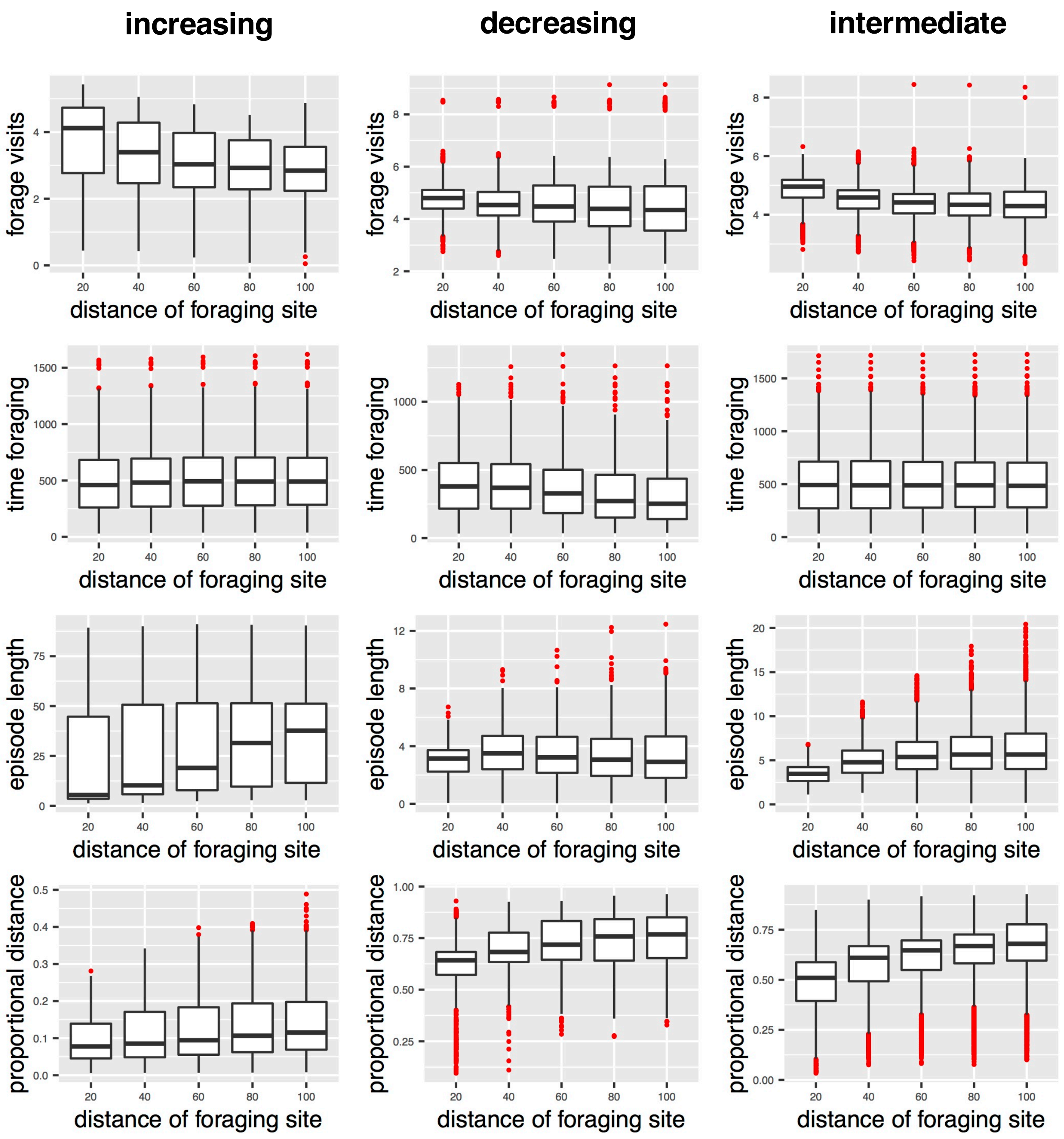

Figure S14. Effects of distance of foraging site on various summary statistics, according to policy form. See panels for details of summary statistic, and see figure S1 for description of boxplots.

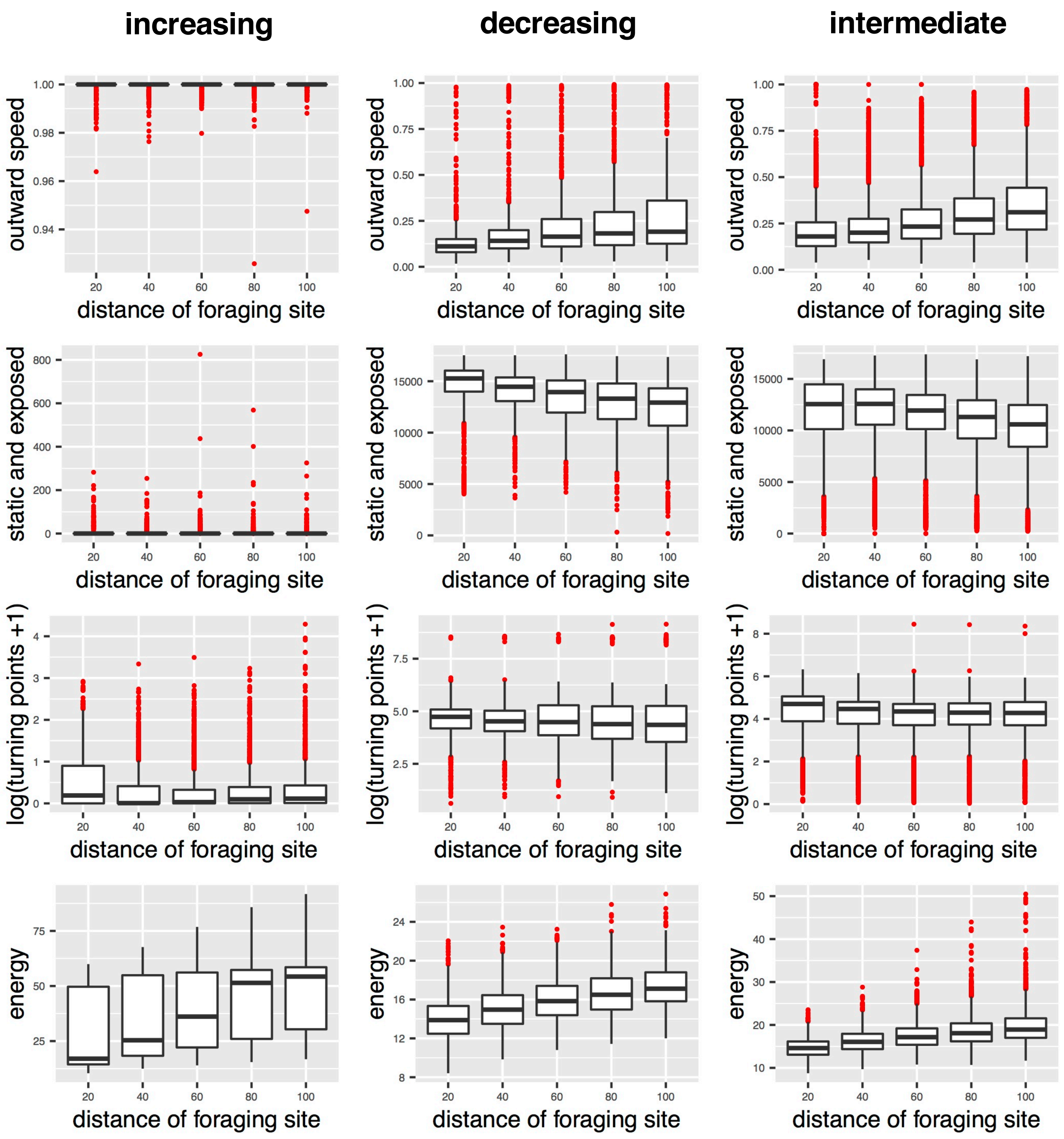

Figure S15. Effects of distance of foraging site on various summary statistics, according to policy form. See panels for details of summary statistic, and see figure S1 for description of boxplots.
